# Determining a Gamma Knife's isocenter using a starburst shot

**DOI:** 10.1002/mp.70434

**Published:** 2026-04-20

**Authors:** Robert Corns, Kainoa Calistro‐Allen, Kaida Yang, Jae Jung, Yanan Cao, Wesley Belcher

**Affiliations:** ^1^ Department of Radiation Oncology, Brody School of Medicine East Carolina University Greenville North Carolina USA; ^2^ Department of Radiology, Heersink School of Medicine University of Alabama at Birmingham Birmingham Alabama USA; ^3^ Department of Radiation Oncology Icahn School of Medicine at Mount Sinai New York City New York USA

**Keywords:** Gamma Knife, isocenter, panoramic, quality assurance, SRS, starburst shot

## Abstract

**Background:**

The Gamma Knife is an important treatment unit for stereotactic radiosurgery. The isocenter is well understood from the collective behavior of all its 192 Co60 sources but is not known from the behavior of individual sources.

**Purpose:**

To introduce and validate the “starburst shot,” a new quality assurance (QA) technique to better understand the Gamma Knife's isocenter by analyzing the convergence of all individual collimator beam axes.

**Methods:**

We built cylindrical phantoms to acquire panoramic radiographic films for 4‐, 8‐, and 16‐mm collimator sizes. We developed an algorithm to locate the centers of each entrance and corresponding exit exposure on the panoramic film, map their centers back to the cylinder, and connect the centers with a line segment in 3D. These lines represent the collimators’ central axes, and we analyzed the resulting cluster of 192 lines using an optimization algorithm to locate the smallest sphere that simultaneously touches all lines. This sphere's center is an estimate of the isocenter, and the radial distances from the isocenter were scored by the probability $p_{0.5}$ of passing within 0.5 mm of the isocenter.

**Results:**

This optimization method provided a robust determination of the isocenter. For the 4‐mm collimators, $p_{0.5}$ was 1.00. For the 8‐mm collimators, $p_{0.5}$ ranged from 0.81 to 0.93, and for the 16‐mm collimators, it ranged from 0.74 to 0.92. The isocenters determined for the 4‐, 8‐, and 16‐mm collimators were found to be within 0.2 mm of each other. In a double‐exposure experiment, the starburst shot accurately found a 1‐mm shift introduced between exposures.

**Conclusions:**

The starburst shot is a new, effective technique for detailed Gamma Knife isocenter QA, providing a comprehensive analysis of all 192 beams. It successfully verifies the isocenter's position and size with submillimeter accuracy. The starburst shot differs significantly from standard techniques, such as the pinprick tool test, and we propose a tiered system of acceptable $p_{0.5}$ values of 0.90 for 4 mm, 0.80 for 8 mm, and 0.70 for 16 mm, as a QA standard for clinical use.

## 1 INTRODUCTION

The Gamma Knife is a remarkable achievement in precision engineering because 192×3(576) independently machined collimators must have their central axes pointing to a single point in space, the isocenter. The starburst shot is a new method to determine properties of the isocenter for a Gamma Knife treatment unit. The isocenter location and size are important quantities to characterize in any stereotactic radiosurgery (SRS) program. The isocenter of the Gamma Knife lies in a 0.25‐mm radius sphere^1^ while for SRS linear accelerators it must lie in a 1 mm diameter sphere.^2^, ^3^

The Gamma Knife's design arranges the sources in 5 sets of rings and groups the sources into 8 sectors with 24 sources per sector. Each sector operates independently of the others and all sources in a sector can be blocked or positioned above 4, 8, or 16 mm collimators. Quality assurance (QA) standards characterize the total radiation field of all 192 sources. The Radiation Focal Point (RFP) is studied with film‐ or detector‐based measurements. The pinprick tool is a film‐based system to compare the RFP to the Unit Center Point (UCP). A small rotatable stage is designed to hold the film on the UCP. The tool makes a pinprick mark on the film that is exactly at the mechanical center (100, 100, and 100 mm) when the tool is irradiated. Exposures are taken on either the xy or xz planes. The radiation center is found by taking a profile through the pin mark and determining the midway point between the two 50% edge doses. Novotny et al.^4^ found the radiation and mechanical centers to be within 0.10 mm of each other, while Task Group 178^1^ recommend a 0.25 mm tolerance level. Diode and diamond detectors have scanned the radiation field in air and in phantom.^1^, ^4^, ^5^ Novotny using the PPS diode tool found the difference between the RFP and UCP to be less than 0.15 mm, while Maraghechi et al.^5^ measured in‐phantom dose profiles with a diamond detector in a spherical phantom. They reported gamma map accuracies of 1%/1 mm for computed versus measured profiles while the computed versus film profiles had gamma map accuracies of 2%/1 mm. The Task Group 178 recommend a 0.2 mm tolerance for the PPS Focus Precision Test—the daily diode‐in‐air scan comparing the RFP against the UCP.

The starburst shot builds up a description of the isocenter by considering individual sources. This is a fundamentally different approach than the techniques that study the total radiation field and will require a different set of concepts for characterizing the isocenter. In addition, new questions can be addressed by the starburst shot such as: “Do the 4‐, 8‐, or 16‐mm collimators share the same isocenter”; “What is the uncertainty in the isocenter”; and “Can we identify misaligned collimators?.”

**FIGURE 1:**
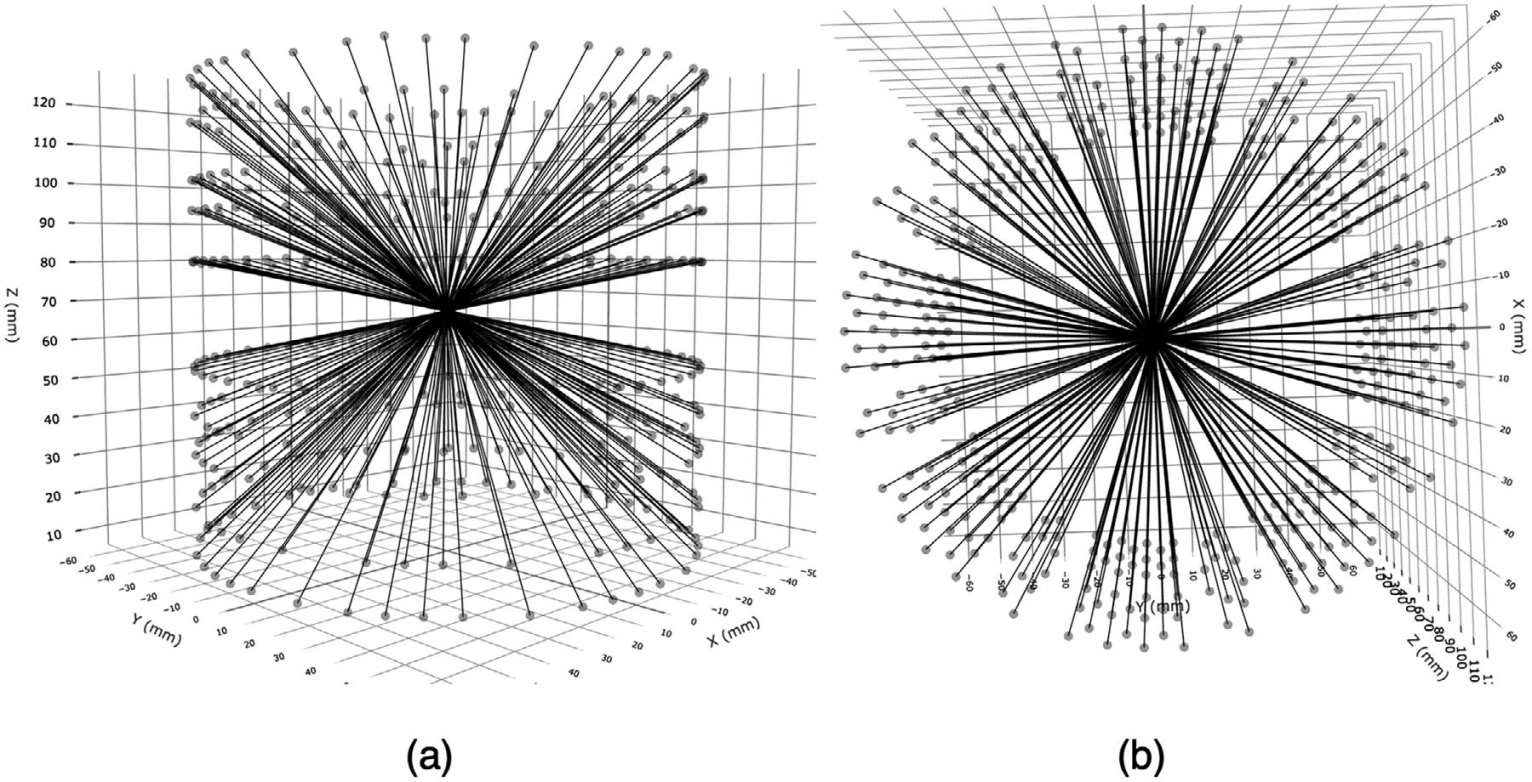
(a) A side view and (b) an axial view of an 8 mm starburst shot. Exposure centers are mapped from the film to the cylinder surface. Each line connecting matching centers represents a collimator axis. The isocenter is defined by where these axes lines converge.

The starburst shot takes its inspiration from two QA techniques: the panoramic film^6^; and the star shot.^7^ The term “starburst shot” was picked as a reference to the original star shot, while visually, it resembles a starburst. Figure 1 shows a typical example.

Cho et al.^6^ were the first group to take a panoramic image on the Perfexion model of the Gamma Knife by exposing a film wrapped around a cylinder. The 192 sources produce a pattern of entrance and exit exposures. Cho sought to confirm all 192 sources were in place and to study the dosimetric properties of the individual exposures.

The starburst shot takes a panoramic film and extracts the centers of all entrance and exit exposures, making 192 pairs of centers. Each center's coordinates is mapped onto the 3D cylinder and then connected dot‐to‐dot to its pair, forming the starburst shot image. The analysis finds the smallest sphere to touch all line segments simultaneously, and the center of this sphere is defined as the isocenter. Once the isocenter is known, the radial distance from the isocenter to each line is computed, and the distribution of the radial distances is analyzed to find the starburst shot's test score.

It is instructive to study similar methods developed for SRS linear accelerators. The Winston‐Lutz method^8^ uses a high‐Z ball‐bearing (BB) positioned at the isocenter of a linear accelerator, and images are taken at different gantry angles. The displacement between the BB's center and the radiation field's center is measured to assess the isocenter's location. The Winston‐Lutz method is the de facto standard for SRS isocenter QA, and forms the basis for many automated variants. It resembles the starburst shot because it considers projective imaging from multiple directions.

More recently, several groups have developed different phantoms and techniques to evaluate linear accelerator isocenters in 3D. Pant et al.^9^ used a gel phantom to obtain a starburst‐like image but with many fewer spokes. Song et al.^10^ have a concept very similar to ours. They wrap a film between a pair of cylinders to capture the entrance and exit exposures of individual spoke shots. The centers of the exposures are mapped from the film onto the cylinder surface and connected in 3D to form a starburst shot.

## 2 METHODS

### 2.1 Phantoms

**FIGURE 2:**
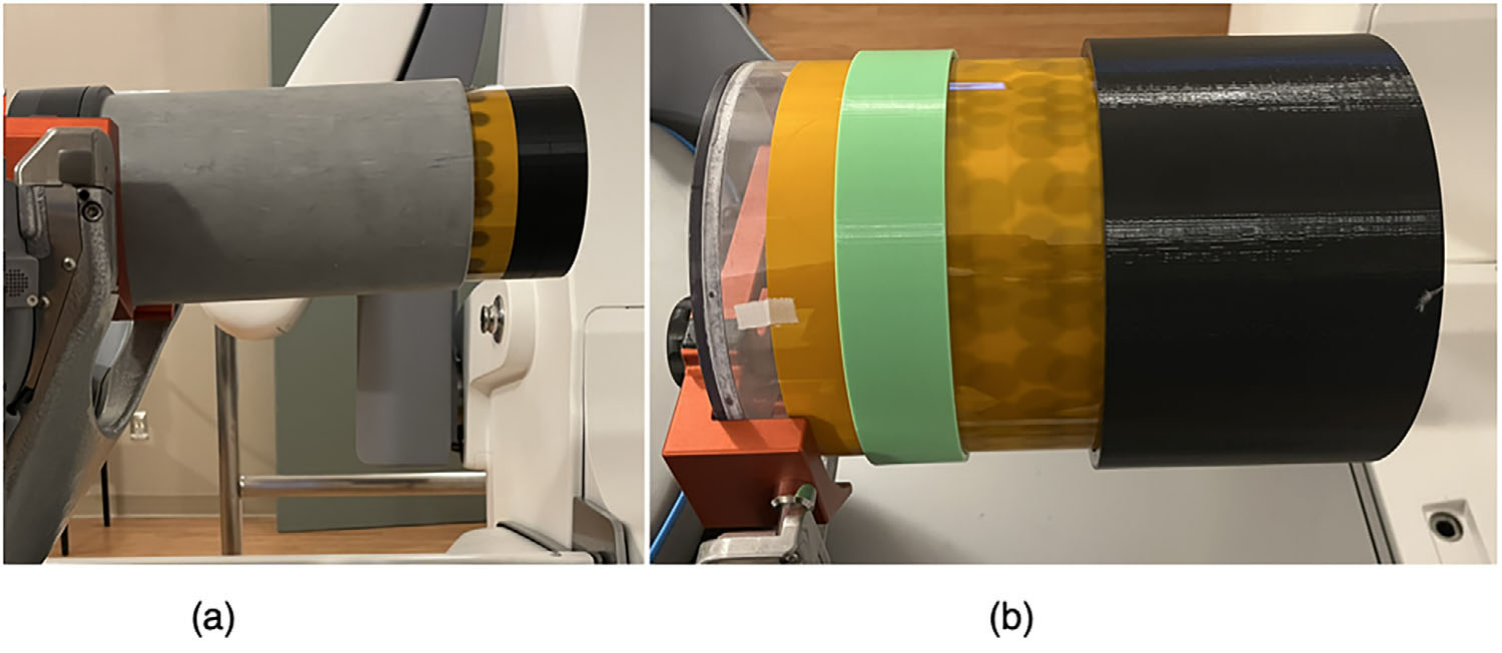
Two cylindrical phantoms were built to take the panoramic images. (a) This is an exploded view of the first phantom, film and buildup assembly. (b) A larger phantom was built to accommodate the 16 mm shots.

Two cylindrical phantoms, shown in Figure 2, were built to take panoramic images. Their dimensions are given in Table 1. The first phantom was made from a PVC electrical conduit and milled into a uniform cylinder. A 17 inch RTQA2 Gafchromic film (Ashland) was able to wrap around the inside of the cylinder with a small overlap. The overlap is important for two reasons: (1) data could be missed if there is a gap and (2) the radius of the cylinder can be determined with high precision from the overlap. Knowing the radius is important because the uncertainty in the isocenter is directly linked to the uncertainty in the radius. This relationship in uncertainties is explored in the Discussion section. Another cylinder was 3D printed to fit snuggly inside the phantom and acts as a buildup layer. An exploded view of the phantom assembly is shown in Figure 2a, where the film and buildup cylinder are pull out from the main cylinder. The phantom has a hanging mount for the Catphan's phantom base plate and leveling screws to ensure it sits parallel to the Gamma Knife's axes.

**TABLE 1:** Cylindrical phantom dimensions.

Phantom	Length (cm)	Inner diameter (cm)	Outer diameter (cm)
Small (PVC)	22.86	12.71	13.65
Large (Plexiglass)	30.48	19.53	20.34

A second, larger cylinder phantom, shown in Figure 2b, was built from Plexiglass. A larger phantom was needed because the 16 mm shot on the small cylinder exhibited too much overlapping of the collimator images. A buildup cylinder was 3D printed to fit snuggly on the outside diameter of the assembled phantom and film.

### 2.2 Phantom centering

**FIGURE 3:**
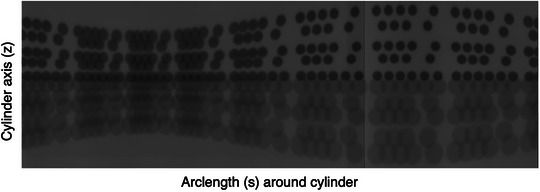
This is a 16 mm panoramic film. A cylinder sitting too far from the Gamma Knife's isocenter produces a wavy pattern in the rows. The pattern contains information on how to shift the cylinder's axis to the isocenter. This shift will straighten the rows and improve the orientation and size uniformity of the ellipses.

The phantoms were not designed to automatically have the mounted cylinder sit perfectly on the isocenter. In retrospect, this would have been a valuable design feature. Consequently, our phantoms can give many details regarding the isocenter's uncertainty and make very exact comparisons between collimator images on the same film, but cannot easily give absolute positioning information. Each cylinder can be leveled to sit parallel to the Gamma Knife's z axis, and the phantoms can be centered on the Gamma Knife by shifting the couch. The small phantom can have a CBCT taken and planned with a shot on its axis. The large phantom will collide with the CBCT scanner and cannot be planned. It can be sent into the Gamma Knife to expose the film, with no shifts in the xy plane. That film, shown in Figure 3, can be used to estimate the required xy‐shift to move the large phantom to the isocenter.

A brief explanation is given here to orient the reader looking at a panoramic image. The circular collimators have nearly elliptical images, and each collimator has an entrance and exit image pair. The rows can be paired by first numbering them as rows 1–10 from top to bottom. Then, radiation entering the cylinder on row 1 will exit at row 10. Likewise, rows 2 and 9 form an entrance/exit pair, as do rows 3 and 8; rows 4 and 7; and rows 5 and 6. Each entrance ellipse has a corresponding exit ellipse located on the opposite side of the cylinder. This means the matching entrance and exit ellipse pairs are half a film apart.

If the cylinder sits far from the isocenter, then the resulting film image will be a wavy arrangement of ellipses. Having the cylinder sitting on the isocenter is preferred. The wave pattern on this film can be used to estimate a shift required to move the cylinder into position for subsequent films. If the cylinder's radius is R, the radial shift from the isocenter is h, and the height (z‐coordinate) on the film for the entrance and corresponding exit exposures are z and $z^{\prime }$, respectively, then

(1) $$h=R\sqrt{1-{\left(\frac{{\left(z-z^{\prime }\right)}_{\min}}{{\left(z-z^{\prime }\right)}_{\max}}\right)}^{2}.}$$

The proof for this formula is provided in Appendix 1.

### 2.3 Film exposures

Once the phantom shift was determined by Equation (1), panoramic films were taken with 30‐min exposure times. We studied the isocenters for 4‐, 8‐, and 16‐mm films. The mixed collimator shots 4 and 8, 4 and 16, and 8 and 16 mm allowed us to study the isocenter of each collimator relative to the others. An Epson Expression 12000XL scanner scanned all films.

### 2.4 Analysis: Overall algorithm

**FIGURE 4:**
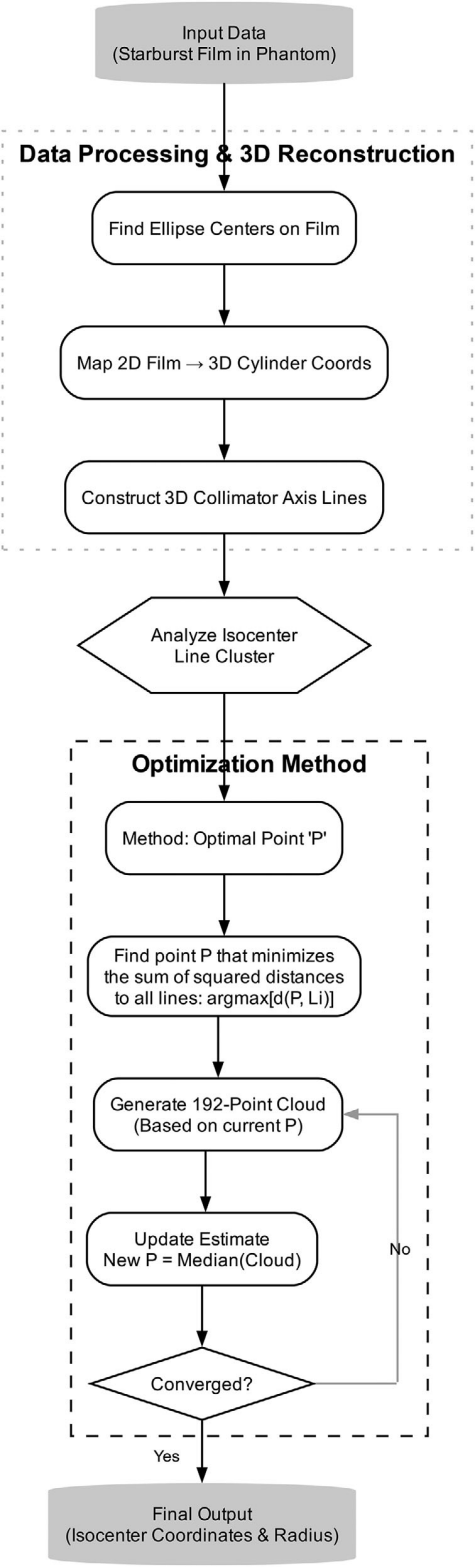
The workflow diagram for finding the isocenter.

**FIGURE 5:**
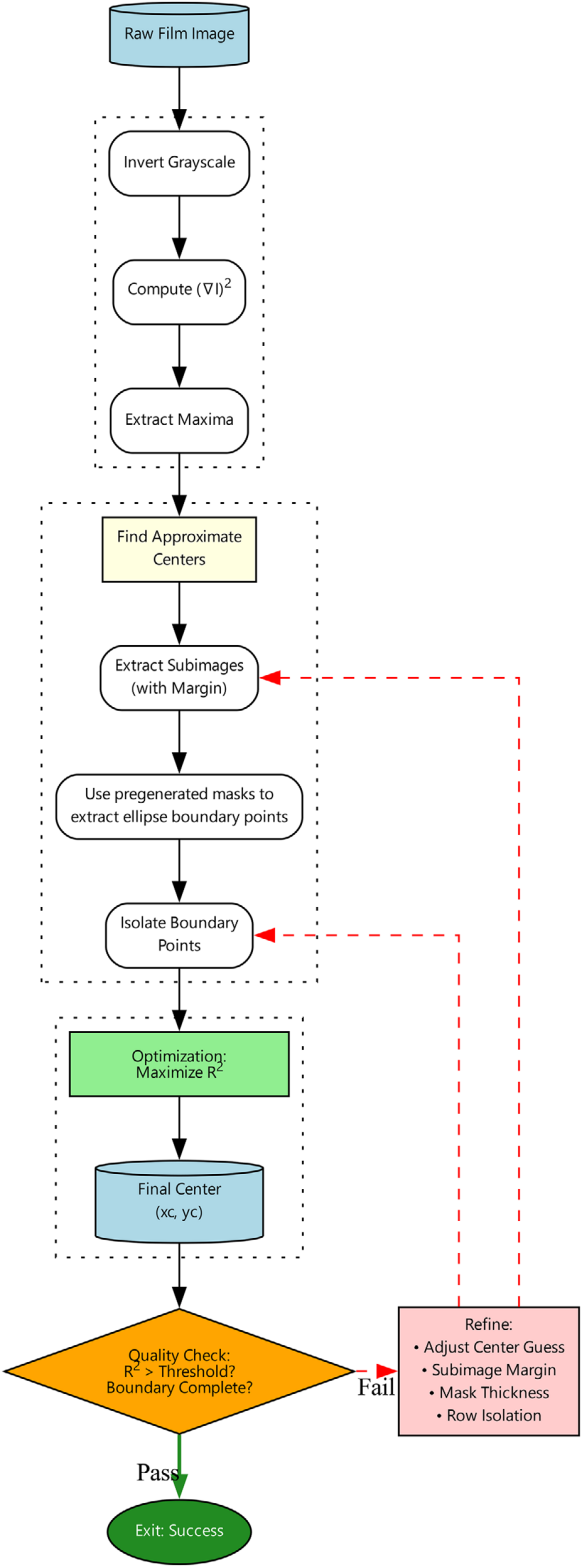
The workflow diagram for finding the center of each exposure ellipse on a film.

The key tasks to analyze a film are outlined in Figure 4. The first step to find the center of each ellipse is a complicated task. This process is illustrated in Figure 5. The centers are mapped to the 3D cylinder surface, where a line segment connecting paired entrance and exit ellipses center‐to‐center represents the collimator axis. Plotting these line segments on a 3D graph forms a starburst shot similar to the one illustrated in Figure 1. The line segments cluster around the isocenter, and the cluster's center is an estimate of the isocenter. The spread of this clustering becomes the final score for the starburst shot test. The computer codes were implemented in R (R 4.5.1, R Foundation for Statistical Computing, Vienna, Austria) together with R's Imager package (0.42.18) and the integrated development environment RStudio (2025.09.2+418, Posit PBC).

Careful examination of the panoramic films shows features about the ellipses that are useful for the analysis. In Figure 3, there is a sinusoidal pattern where the ellipses on a given row have subtle changes in size and orientation. Compare this to Figure 8, where the rows are much straighter and the ellipses on a given row are of the same size and have a vertical semimajor axis. The uniformity of the ellipses simplifies the analysis because we need only consider fitting 10 types of ellipses instead of fitting 384 ellipses.

### 2.5 Analysis: Finding the edge points

**FIGURE 6:**
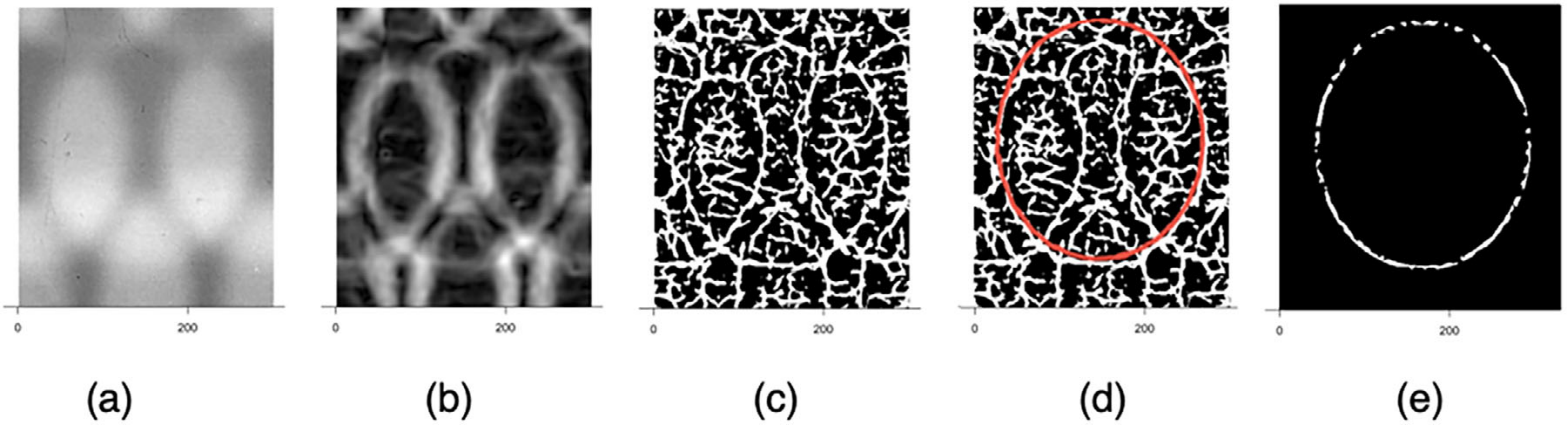
Finding the ellipse edge in a small sub‐image follows these steps: (a) Invert the film's gray scale to get image I; (b) Take the square of the gradient of the image, ${\left|\nabla I\right|}^{2}$; (c) Find the maxima in ${\left|\nabla I\right|}^{2}$; (d) Move a mask (in red) around to score the maximum overlap with the white pixels; and (e) Use a combination of the mask and morphological operations to extract points from the ellipse's edge.

An ellipse's edge points need to be identified to find its center. The edge is located at the local maxima of the modulo square of the image's gradient.^11^ Figure 6 shows a pictorial sequence of the steps to find the edge points of one ellipse.

First, the film image is inverted to image I in Figure 6a. A fast edge‐detection routine was developed to find the ellipse edges from I. The Imager package in R takes the gradient of image I and squares it to form an image ${\left|\nabla I\right|}^{2}$, as shown in Figure 6b. This image depicts the edges of the ellipse as bright bands, but there are several issues with these bands, such as bands from adjacent ellipses confounding the analysis. The ellipse edges occur at the local maxima of this image, and an efficient algorithm to find local maxima was developed. Figure 6c shows the resulting binary image ${{\left|\nabla I\right|}^{2}}_{\max}$. Since the maxima algorithm is fast but sensitive to noise, a mask was used to select the boundary points of the ellipse. Ten different masks, one per row, were created by fitting a single ellipse on each row. The fitted function was transformed into an image mask by plotting it into a blank sub‐image. The idea is to move the mask around the ${{\left|\nabla I\right|}^{2}}_{\max}$ image and score the number of white pixels under the mask. The maximum score occurs when the mask sits over the ellipse edge points. Figure 6d shows a red mask that is positioned slightly to the side of the ellipse. The thickness of the line in the mask can be increased to improve its sensitivity to capturing the ellipse's edge points. Once the location of for the maximum score is found, pixels to the ellipse's edge are selected by performing an AND operation between the mask and ${{\left|\nabla I\right|}^{2}}_{\max}$ and that selection is expanded using morphological operations. The final selection is shown in Figure 6e.

### 2.6 Analysis: Finding the center

The ellipse edge points are used to find the location of the ellipse's center by considering the equation of an ellipse:

(2) $$\frac{{\left(x-P_{x}\right)}^{2}}{a^{2}}+\frac{{\left(y-P_{y}\right)}^{2}}{b^{2}}=1.$$

This equation is applicable to any ellipse whose major and minor axes are parallel to the x and y axes. The point $\left(P_{x},\;P_{y}\right)$ is the center of the ellipse.

Now consider a data set where (x,y) is a boundary point of an ellipse whose center is $\left(x_{c},\;y_{c}\right)$. Treat the point $P=(P_{x},P_{y})$ as a parameter and transform each point (x,y) from the boundary set to

(3) $$\;\left(X,\;Y\right)=\left({\left(x-P_{x}\right)}^{2},\;{\left(y-P_{y}\right)}^{2}\right).\;$$

The quadratic form in Equation (2) can be rearranged into the following equation:

(4) $$Y=-\frac{b^{2}}{a^{2}}X+b^{2}.$$

**FIGURE 7:**
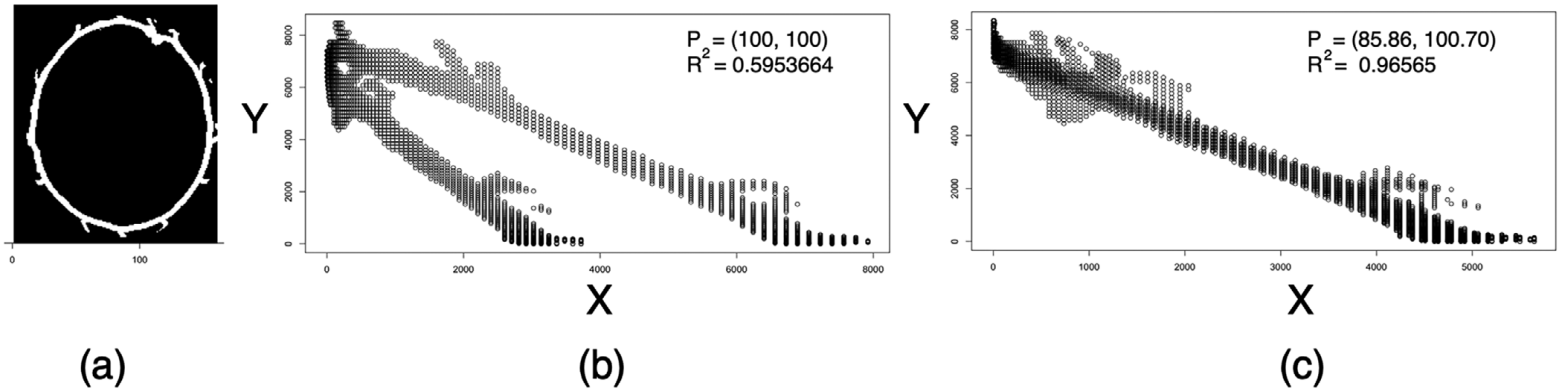
Finding the center of the ellipse by moving P to find the maximum $R^{2}$ value. (a) The edge points of the ellipse are used to generate (X,Y). (b) A trial guess of P = (100, 100) produces this graph with $R^{2}=0.595.$(c) The optimal point P=(85.85,100.70) results in $R^{2}=0.966$ and the graph of (X,Y) is linear.

While Equation (4) appears to be a linear equation, the graph of (X,Y) is not, unless the point $P=(x_{c},\;y_{c})$. Finding the center becomes an optimization problem to move the point P with the objective of maximizing the coefficient of determination ($R^{2}$) between Y and X. Figure 7 illustrates the optimization concept by first using a generic point P=(100,100) and then the optimal point P.

This technique does not require a complete ellipse edge set, which is useful when the ellipse has been cut off by the side of the film. However, it does require a well‐defined ellipse edge set.

### 2.7 Analysis: Creating a starburst shot

The ellipse centers on film have coordinates (s,z), where s is the arclength along the cylinder surface and z is the length along the cylinder axis. The origin for the z‐axis is typically defined from the edge of the film image. That means the z coordinate of the isocenter is related to the size of the film. The arclength can be converted to (x,y) in the cylinder's coordinate system by the following equations:

(3) $$x=R\sin\left(\frac{s}{R}-\varphi \right)$$

(4) $$y=R\cos\left(\frac{s}{R}-\varphi \right),$$

where R is the radius of the cylinder and φ is a phase shift that represents the point from where the edge of the film begins on the cylinder relative to the top of the cylinder. The starburst shot is formed by connecting the center of an entrance ellipse to its corresponding exit ellipse in the 3D space.

### 2.8 Analysis: Finding the isocenter

The isocenter is found by locating the smallest sphere to touch all line segments in the starburst shot. This can be accomplished as an optimization problem followed by a reassignment of values. Let $\left\{a_{1},\;a_{2},\text{…},a_{192}\right\}$ be the set of line segments forming the starburst shot in $R^{3}$ and let $P\in R^{3}$ be an optimization point. There is a unique point $Q_{k}$ on $a_{k}$ that is closest to the point P. Moreover, as P moves around so do the $Q_{k}$’s. The optimization problem is expressed as: $\mathrm{argmi}n_{P}\left(\max_{k}\left|P-Q_{k}\right|\right)$. The resulting value for P is the first estimate of the isocenter in the cylinder's coordinates. This optimal point P has the unfortunate property of relying on the most extreme $|P-Q_{k}|$ distance. If you plot the points P and $\left\{Q_{1},\;Q_{2},\;\text{…},Q_{192}\right\}$ in a 3D plot, the point P may not look centered in the point cloud. A final estimate for the isocenter is made by reassigning P to the median position of the $Q_{k}$’s, and since the $Q_{k}$ depend on P, these were recalculated. These new values reduce most of the distances $|P-Q_{k}|$ at the expense of a few of the distances increasing.

Ideally, if the cylinder is well placed, then the isocenter is $P\;=(0,\;0,\;z_{0})$, but in practice, $P_{x}$ and $P_{y}$ are close to 0 while $P_{z}$ is a coordinate relative to the film edge. More importantly, the distribution of distances $|P_{\mathrm{iso}}-Q_{k}|$ can be found. The final score for the starburst shot test is derived from this distribution. A different standard is needed than what has been used for the Gamma Knife and for linear accelerators.

### 2.9 Analysis: Starburst shot score

The starburst shot does not follow standard analysis used in either GK or linear accelerators and requires a new approach which we cover in detail in the Discussion. In short, we propose that the probability a given collimator's axis is within 0.5 mm of the isocenter is an appropriate quantity to monitor. Denote this probability by $p_{0.5}$. The probability is estimated by looking at the fraction of sources whose distance to the isocenter is ≤0.5 mm. Ideally, $p_{0.5}=1$ but in practice, $p_{0.5}<1$.

### 2.10 Verification

The starburst shot is a new technique without a comparative standard to verify its results. Ideally, a well‐designed phantom would sit naturally on the mechanical isocenter and provide a connection between the RFP and UCP. Our phantom was not designed to this specification. We can accurately assess the uncertainty in the isocenter, and we can accurately compare isocenters of sectors on the same film. This second property means that we can simulate a situation where not all the sources appear to be pointing to a single center. The mechanical accuracy of the couch provides a gold standard for comparison.

Consider a double exposure. The sectors will be either 4 mm or blocked. The first exposure blocks sector 1, while the others are open. This shot is taken at the isocenter. The second exposure has sector 1 open, while blocking the other sectors. This shot is shifted 1 mm from the isocenter along the y axis.

This double exposure can be used in two experiments. (1) The pinprick tool test checks the total radiation field's center. We predict the radiation center will not be shifted more than 0.125 mm from the pinprick center even if 12.5% of the sources are shifted by 1 mm. A control film will be single exposure film taken at the isocenter with all sectors open. The double exposure film is taken as described above.

(2) The double exposure on a starburst shot should show two distinct clusters of points separated by 1 mm.

## 3 RESULTS

### 3.1 Cylinder shift

Equation 1 is used to estimate the shift required to move the isocenter to the central axis of the large phantom. The phantom can be centered on the Catphan's base plate and hence the shift is only along the y axis. Table 2 shows the minimum and maximum differences in $z-z^{\prime }$ and the resulting calculated shift for each pair of entrance and exit rows. We expect the row pair 1 and 10 to have the most accurate estimate. We take an image using the 30.3 mm shift along the y axis to estimate the isocenter position. Combining the isocenter's position with the 30.3 mm shift we get our standard shift for subsequent films.

**TABLE 2:** Estimates for the cylinder shift from the film in Figure 3.

Row pair	${\left(z-z^{\prime }\right)}_{\min}$ (pixels)	${\left(z-z^{\prime }\right)}_{\max}$ (pixels)	Shift h (mm)
1 & 10	1730	1651	30.3
2 & 9	1345	1266	34.4
3 & 8	858	811	33.3
4 & 7	558	503	44.2
5 & 6	148	109	68.8

The final shift we settled on for the large phantom was 23.2 mm along the *y* axis. We arrived at this iteratively by taking a film, computing the shifts and then taking a new film and computing its shift. We would stop once the rows have straightened.

### 3.2 Panoramic films

**FIGURE 8:**
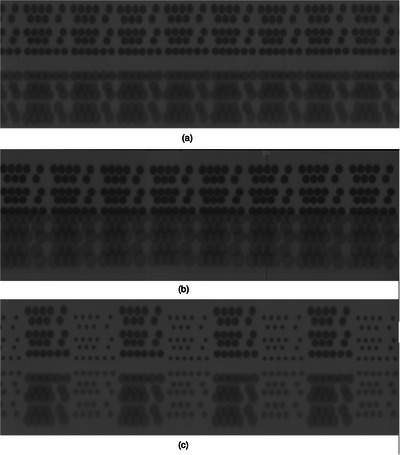
(a) 8 mm collimators taken on the small phantom. (b) 16 mm collimators taken on the large phantom. (c) A mixture of 4 and 8 mm collimators, taken on the small phantom.

With the issue of shifting the cylinder settled, six different collimator arrangements were studied: 4, 8, and 16 mm, 4 and 8 mm, 4 and 16 mm, and 8 and 16 mm. Figure 8 shows three of the films we studied to give a sense of what films look like for (a) the small phantom; (b) the large phantom; and (c) how different sized collimators appear on one film.

The software finds each ellipse center on film, maps these to the cylinder surface, and creates a starburst shot. Comparing isocenters between films is a comparison between the reproducibility of the cylinder setup. With the current cylinder design, the only true isocenter comparisons that can be done are between mixed collimators taken on a single film.

Table 3 shows the isocenter results for the films. The isocenter coordinates are $\left(x_{\mathrm{iso}},y_{\mathrm{iso}},z_{\mathrm{iso}}\right)$. The $x_{\mathrm{iso}}$ and $y_{\mathrm{iso}}$ values are a reflection on how close the cylinder's axis was positioned relative to the isocenter. The $z_{\mathrm{iso}}$ is a statement about where the isocenter sits relative to the edge of the film. The $r_{\mathrm{median}}$ is the median distance the collimator axes are from the isocenter, a useful estimate for probability models.

**TABLE 3:** Isocenter localization result. Isocenters for collimators taken on the same film can be directly compared. The isocenter has coordinates $\left(x_{\mathrm{iso}},y_{\mathrm{iso}},z_{\mathrm{iso}}\right)$. The distribution of distances between the collimator axes and the isocenter are characterized by the median distance $r_{\mathrm{median}}$ and the probability $p_{0.5}$ the distance is ≤0.5mm.

		Optimal point (mm)					
Film	Collimator	$x_{\mathrm{iso}}$	$y_{\mathrm{iso}}$	$z_{\mathrm{iso}}$	$r_{\mathrm{median}}$	$p_{0.5}$	Distance between isocenters (mm)
4 mm	4	−0.22	−0.72	60.53	0.166	1.00	
8 mm	8	−0.18	0.10	67.31	0.292	0.82	
16 mm	16	−1.89	0.76	130.37	0.266	0.90	
4 and 8 mm	4	0.17	0.36	70.76	0.168	1.00	0.10
	8	0.09	0.32	70.72	0.264	0.81	
4 and 16 mm	4	−1.16	−0.68	92.70	0.127	1.00	0.17
	16	−1.25	−0.68	92.84	0.223	0.92	
8 and 16 mm	8	1.77	0.73	127.77	0.238	0.93	0.12
	16	1.78	0.70	128.12	0.322	0.74	

Table 4 shows the results for the pinprick tool test. Gafchromic™ EBT3 film (Ashland) was used for both control and the double‐exposure films. Films were scanned at 300 dpi. The color channels were split and the red channel was analyzed.

**TABLE 4:** Pinprick test results for a control film taken with one exposure of all sectors and a double exposure film where sectors 2–8 expose the UCP in the first exposure and sector 1 exposes the film after a 1 mm shift along the y axis.

Collimator	Film	Axis	Image size (mm)	Radiation center (mm)	Pin prick center (mm)	RFP—UCP (mm)
4 mm	Control	x	6.18	3.09	3.12	−0.03
		y	6.86	3.43	3.43	0.00
	1 mm shift	x	6.18	3.09	3.12	−0.03
		y	6.77	3.39	3.46	−0.07

**FIGURE 9:**
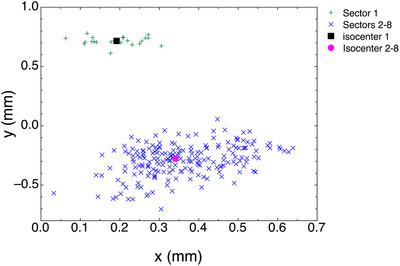
A double‐exposure starburst shot plotting data from sector 1 versus sectors 2 to 8. The sector 1 exposure was shifted 1 mm along the y axis from the sectors 2 to 8 exposures.

Table 5 has the starburst shot results for the double exposure. Figure 9 is a graph for the starburst double exposure. While technically a 3D problem, the 2D graph in Figure 9 demonstrates the result.

**TABLE 5:** Double exposure on a starburst film. Sectors 2–8 expose the UCP in the first exposure and sector 1 exposes the film after a 1 mm shift along the y axis.

	Isocenter			
Sector	x (mm)	y (mm)	z (mm)	Radial difference (mm)
1	0.192	0.716	81.421	
2–8	0.341	−0.276	81.704	
Difference	−0.149	0.992	−0.283	1.043

## 4 DISCUSSION

**FIGURE 10:**
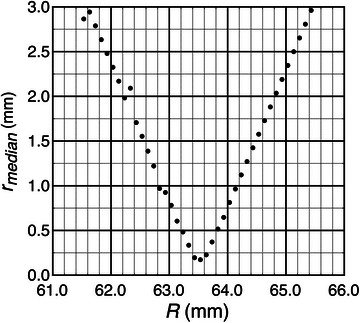
When treating the cylinder radius R as a parameter, an optimal value gives a minimum to the isocenter radius $r_{\mathrm{median}}$. This optimal value corresponds with the physical cylinder radius.

A key reportable quantity is the uncertainty of the isocenter's location, as characterized by the median radius $r_{\mathrm{median}}$. Unexpectedly, the cylinder's radius R directly affects this quantity. The difficulty lies in the conversion of the arc length, s, into (x,y) coordinates relying on R. The relationship between $r_{\mathrm{median}}$ and R can be explored by treating R as a parameter, then remapping s into (x,y), and seeing the impact on $r_{\mathrm{median}}$. Figure 10 is a plot showing the results from one of these simulations. The graph shows $r_{\mathrm{median}}$ changes 1.5 mm for every 1 mm change in R. If we want to know $r_{\mathrm{median}}$ to within 0.1 mm, then we will need to know R to within 0.07 mm.

One way to determine the radius of the cylinder with submillimeter accuracy is to use the double image created where the film overlaps. The centers of duplicated ellipses appearing on the left and right side of the film will be one circumference apart. This distance is known to the pixel. For example, the 4 mm shots in Figure 8c have duplicated ellipses that are 4717 pixels apart. Since the film was scanned at 300 dpi, the cylinder radius is 63.56 mm.

The probability $p_{0.5}$ is of interest because it ties this work to the 0.5 mm standard for SRS linear accelerators. The 4 mm collimator score is $p_{0.5}=1.00$ in each of the 4 mm films. The 8 mm collimator scores range from 0.81 to 0.93 and the 16 mm collimator scores range from 0.74 to 0.92. This suggests a tiered system depending on the collimator for accepting the starburst shot test. While the 4 mm collimators had perfect scores, we recommend allowing some leeway for variability not observed in our films. The 8 and 16 mm collimators while not perfect had respectably high probabilities. We recommend giving a slightly lower threshold for acceptable results. Table 6 is a suggested scheme for evaluating the starburst shot.

**TABLE 6:** A criterion for determining if a Gamma Knife unit passes a starburst shot QA measurement.

Collimator	$p_{0.5}$ Threshold
4 mm	0.90
8 mm	0.80
16 mm	0.70

Since the Gamma Knife is described as having a 0.3 mm accuracy, what is the value in having a 0.5 mm standard? First, the comparisons used for the 0.3 mm value are not directly comparable for the starburst shot. This is because the existing standards apply to the total radiation field, while the starburst shot works with individual source fields. This effect was demonstrated with the double‐exposure experiment. The pinprick result for the double exposure film was an additional 0.07 mm shift in comparison to the control film. Based solely on the double‐exposure film, this value is not alarming, and one would conclude there was no problem with the total radiation field. But we are aware of the 1 mm shift, and this implies the pinprick standard was incapable of detecting this shift. This fact should not be too surprising because we are comparing the impact of 24 sources against 168 stationary sources. When we imagine this as a problem in weighted averages, we predicted a shift on the order of 0.125 mm. This is in reasonable agreement with the observed 0.07 mm. If we cannot rely on our usual standards for the starburst shot, what values are reasonable? The linear accelerators use a 1 mm diameter standard for SRS and effectively, starburst shots are being done on linear accelerators. However, this is also with many fewer exposures than the 192 exposures of Gamma Knife. It made sense to use a 0.5 mm threshold but with some modification to account for the larger sample size.

We measured axes further than 0.5 mm from the isocenter. It is difficult to decide between the following causes for these deviations. They may be due to inaccurate construction; they could be due to random deviations in the data; or they could be systematic errors from our algorithms. One approach to determine which possibility is correct would be to track each source's radial values over multiple repetitions. A random effect would mean different sources would exhibit these extreme distances. Systemic or construction errors would on the other hand would flag the same source as being consistently further. Systemic errors could be caught by careful reviews of the algorithms or by changing to independent algorithms that achieve results through different methods.

Verifying our results was difficult because our cylinders were not designed to give absolute positioning relative to the Gamma Knife's UCP. Rather, our cylinders were designed to measure the radial uncertainty in the isocenter and determine the relative positioning of each sector's isocenter with high accuracy. This suggested a possible verification method. The double‐exposure technique would shift one sector relative to the others between exposures. The shift is accomplished by moving the couch. Confirming that the measured shift equals the couch shift is the verifying evidence for the starburst shot. Table 5 shows the isocenter shift along the y axis to be 0.992 mm with an overall radial shift of 1.043 mm. This is in excellent agreement with the 1.0 mm couch shift along the y axis. Figure 9 supports this result graphically, plotting the 192 $Q_{k}$ points together with the centers P of each point cloud.

## 5 CONCLUSIONS

The starburst shot is a new technique for finding the isocenter of a Gamma Knife by studying the individual behavior of each source. The reportable quantity is the probability $p_{0.5}$, which is the likelihood a collimator axis is within 0.5 mm of the isocenter. We propose a tiered system for passing a starburst shot of $p_{0.5}=0.90$ for the 4 mm collimator, 0.80 for the 8 mm collimator and 0.70 for the 16 mm collimator.

The starburst shot may also be used to compare radiation‐field centers with high accuracy, provided the fields expose the same film. We found differences between the isocenters for 4, 8 and 16 collimators to be less than 0.2 mm. Additionally, with the double exposure experiment, the starburst shot accurately tracked the 1.0 mm couch displacement made for sector 1′s exposure. The same double exposure on the pinprick tool test did not show an appreciable shift in the radiation center. This clearly demonstrates a difference between the individual radiation fields and the total radiation field. Consequently, new standards need to be developed for methods, such as the starburst shot, that study individual sources.

The starburst shot is an exciting technique that opens the door for many new possibilities in the Gamma Knife's QA program, ultimately improving patient care.

## CONFLICT OF INTEREST STATEMENT

The authors declare no conflicts of interest.

## Figures and Tables

**FIGURE A1:**
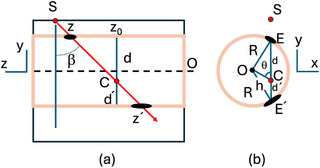
The wavy pattern of ellipses on the panoramic film is a consequence of the cylinder being offset some distance h from the isocenter C. The source S shoots radiation through the isocenter and has entry E and exit E´points on the cylinder. (a) A section view through the plane of the isocenter. (b) An axial cut through the isocenter showing the offset h from the center of the cylinder to the isocenter.

## APPENDIX 1

The wavy pattern in Figure 3 is due to an offset h of the cylinder's central axis from the isocenter. This film is more challenging to analyze because of the slow changes along a row in the ellipse orientation and size. A simple model can estimate h. Using this value of h to move the cylinder's axis closer to the isocenter straightens the rows and makes the ellipses more uniform in size and orientation.

The geometry is shown in Figure A1 with two orthogonal views. The cylinder axis is labeled by O and the cylinder has radius R. In the side profile picture, O is off plane. The isocenter is C with z axis coordinate $z_{0}$ and the cylinder has a radial offset h from the isocenter in some direction. When looking at a given source S, this direction is parameterized by angle θ. The cylinder edge will be a distance d(θ) above the isocenter. The source S shoots through the isocenter, and the radiation enters the cylinder at point E with coordinate z and exits at E´ with coordinate $z^{\prime }$. The incident angle of the radiation is β. The relation between z and d is

(A1)
$$z-\;z_{0}=d\tan\beta$$

Looking at the upper triangle OCE in Figure A1.b, by the law of cosines

(A2)
$$\;R^{2}=h^{2}+d^{2}-2hd\cos\theta$$

This is a quadratic in d that can be solved but only the positive root is physical:

(A3)
$$\begin{array}{cc} d & =\frac{2h\cos\theta +\sqrt{4\;h^{2}\cos^{2}\theta -4\left(h^{2}-R^{2}\right)}}{2}\\ & \;=h\cos\theta +\sqrt{R^{2}-h^{2}\sin^{2}\theta } \end{array}$$

Combining Equations (A1) and (A3),

(A4)
$$z=z_{0}+\tan\beta \left(h\cos\theta +\sqrt{R^{2}-h^{2}\sin^{2}\theta }\right)$$

It is also possible to solve for the exit location. A similar argument uses the primed variables and replaces θ by π−θ:

(A5)
$$\;d^{\prime }=-h\cos\theta +\sqrt{R^{2}-h^{2}\sin^{2}\theta }$$

and

(A6)
$$z^{\prime }=z_{0}\;-\tan\beta \left(-h\cos\theta +\sqrt{R^{2}-h^{2}\sin^{2}\theta }\right)$$

Equations (A4) and (A6) are useful because they allow us to eliminate $\;z_{0}$:

(A7)
$$z-\;z^{\prime }=2\tan\beta \sqrt{R^{2}-h^{2}\sin^{2}\theta }$$

Looking at the minimum and maximum values of $z-z^{\prime }$, we can eliminate tanβ and solve for h to get Equation (1):

(A8)
$$\;{\left(z-z^{\prime }\right)}_{\max}=2R\;\tan\;\beta \;$$

(A9)
$$\;{\left(z-z^{\prime }\right)}_{\min}=2\tan\beta \;\;\sqrt{R^{2}-h^{2}}$$

(A10)
$$h=R\sqrt{1-{\left(\frac{{\left(z-z^{\prime }\right)}_{\min}}{{\left(z-z^{\prime }\right)}_{\max}}\right)}^{2}}$$
